# Occurrence of extended-spectrum beta-lactamase-producing *Enterobacteriaceae,* microbial loads, and endotoxin levels in dust from laying hen houses in Egypt

**DOI:** 10.1186/s12917-020-02510-4

**Published:** 2020-08-24

**Authors:** Marwa F. E. Ahmed, Hazem Ramadan, Diana Seinige, Corinna Kehrenberg, Amr Abd El-Wahab, Nina Volkmann, Nicole Kemper, Jochen Schulz

**Affiliations:** 1 Institute for Animal Hygiene, Animal Welfare and Farm Animal Behaviour, University of Veterinary Medicine Hannover, Foundation, Hannover, Germany; 2 Hygiene and Zoonoses Department, Faculty of Veterinary Medicine, Mansoura University, Mansoura, Egypt; 3 Institute for Food Quality and Food Safety, University of Veterinary Medicine Hannover, Foundation, Hannover, Germany; 4 Institute for Veterinary Food Science, Justus-Liebig-University Giessen, Giessen, Germany; 5 Nutrition and Nutritional Deficiency Diseases Department, Faculty of Veterinary Medicine, Mansoura University, Mansoura, Egypt

**Keywords:** Laying hens, Settled dust, Bacteria, Fungi, ESBL, *Enterobacteriaceae*, Endotoxins

## Abstract

**Background:**

Poultry houses are often highly contaminated with dust, which might contain considerable amounts of microorganisms and endotoxins. The concentrations of microorganisms and endotoxins in dust from laying hen houses in Egypt are unknown. However, to estimate the risks for birds, the environment, and people working in laying hen houses, it is important to gather information about the composition of these dusts. Here we report the microbial loads, the occurrence of antimicrobial-resistant bacteria, and endotoxin concentrations in dust samples from 28 laying hen farms in Dakahliya Governorate, Egypt, and discuss the results relevant to the literature.

**Results:**

Pooled settled dust samples (*n* = 28) were analyzed for total viable counts of bacteria and fungi (CFU/g), the occurrence of extended-spectrum beta-lactamase (ESBL)-producing *Enterobacteriaceae*, *Salmonella* spp., and methicillin-resistant *Staphylococcus aureus* (MRSA), and endotoxin concentrations (ng/g). The means and standard deviations of total viable counts were 7.10 × 10^8^ ± 2.55 × 10^9^ CFU/g for bacteria and 5.37 × 10^6^ ± 7.26 × 10^6^ CFU/g for fungi. Endotoxin levels varied from 2.9 × 10^4^ to 6.27 × 10^5^ ng/g. None of the tested samples contained *Salmonella* spp. or MRSA. In contrast, by direct plating, *Enterobacteriaceae* were found frequently (57%; *n* = 16), and suspected ESBL-producing *Enterobacteriaceae* occurred in 21% (*n* = 6) of the sampled barns. Using an enrichment method, the detection of *Enterobacteriaceae* and suspected ESBL-producing *Enterobacteriaceae* increased to 20 and 16 positive barns, respectively. Taking results from both methods into account, *Enterobacteriaceae* and suspected ESBL-producing *Enterobacteriaceae* were detected in 23 barns Overall, 100 ESBL suspected isolates (*Escherichia coli*, *n* = 64; *Enterobacter cloacae*, *n* = 20; and *Klebsiella pneumoniae n* = 16) were identified to species level by MALDI-TOF MS. Isolates from 20 barns (71% positive barns) were confirmed as ESBL producing *Enterobacteriaceae* by the broth microdilution test.

**Conclusions:**

Dust in Egyptian laying hen houses contains high concentrations of microorganisms and endotoxins, which might impair the health of birds and farmers when inhaled. Furthermore, laying hens in Egypt seem to be a reservoir for ESBL-producing *Enterobacteriaceae*. Thus, farmers are at risk of exposure to ESBL-producing bacteria, and colonized hens might transmit these bacteria into the food chain.

## Background

The occurrence of organic dust particles on poultry farms, which are formed of both viable and non-viable particles, can pose a serious risk to bird production and stockmen health [1]. Airborne dust or settled dust from surfaces in livestock buildings could contain plant particles, particles from food, fecal particles, epithelia, bacterial cells and spores, fungi and fungal spores, viruses, endotoxins, mycotoxins, and antibiotic agents [2]. The amount and the composition of particles in poultry houses are affected by factors such as housed species, stocking density, age and fattening period of the birds, feeding system, antibiotic treatment, bedding material, humidity, ventilation system, and application of hygienic measures [3, 4]. Respiratory diseases of both humans and animals have mainly multifactorial causes, and the quantity and quality of airborne dust play an important role [5]. Dust in poultry houses, for instance, can contain several inflammatory agents, such as endotoxins and (1–3)-ß-D-glucan [6]. Endotoxins are lipopolysaccharides of Gram-negative bacteria that can induce an inflammatory response in humans after inhalation [7]. High endotoxin burdens can also be associated with inflammations and infections of animals [8]. Furthermore, dust in poultry houses can act as a reservoir for zoonotic agents and resistant bacteria. Zoonotic *Salmonella* species (spp.) can occur and survive in dust from laying hen houses, and they can be transmitted horizontally via an airborne route [9]. This might also enhance the spread within a flock, and that can be associated with a higher risk of contaminated eggs [10]. Another risk is the transmission of potentially harmful microorganisms from animals to farmers. For instance, farmers who frequently come into contact with livestock-associated methicillin-resistant *Staphylococcus aureus* (LA-MRSA) have a higher risk of becoming colonized with these bacteria than those persons having no contact with farm animals [11]. Direct contact with colonized animals and frequent contact with contaminated surfaces and inhaling contaminated air are assumed to contribute to nasal colonization [11, 12]. Livestock-associated MRSA isolates harbor the *mecA* gene, which encodes a product that confers resistance to ß-lactam antibiotics [13]. Therefore, screening of dust samples, which is an established method for investigating the LA-MRSA occurrence on farms, can contribute to estimating the risk for farmers of becoming colonized by resistant pathogens. Besides MRSA, extended-spectrum β-lactamase (ESBL)-producing bacteria were discovered worldwide in poultry [14, 15]. These bacteria secrete enzymes, β-lactamases, which confer resistance to β-lactam antimicrobials, including penicillins, cephalosporins, and monobactams [16]. These β-lactamases are often located on extra-chromosomal mobile genetic elements, such as plasmids [17]. Since plasmids harboring resistance genes can be transferred between bacteria of the same species or different genera, such exchanges increase the development of reservoirs of resistance genes in animals and the environment [18]. The role of dust as a source of ESBL-producing bacteria is unknown. Farmers might be colonized and become carriers [19]. Dust can also be a potential long-term reservoir. For example, a recent study showed that resistant *Escherichia coli* (*E. coli*) are able to survive for a considerable period of time in dust from livestock buildings [20].

The studies addressing dust in animal houses have been summarized in a review from Zhao et al. [21]. To our knowledge, there is no information available in the scientific literature concerning viable and non-viable particles in dust from laying hen farms in Egypt. To assess the burden of settled dust from Egyptian laying hen houses with microbial loads, zoonotic agents, resistant bacteria (ESBL producers, MRSA), and endotoxin contents in dust samples from 28 laying hen farms were investigated. Since most studies about farm dust components were conducted in temperate zones [22], our research also aimed to gain more information on the situation in ventilated barns under arid conditions.

## Results

**Fig. 1:**
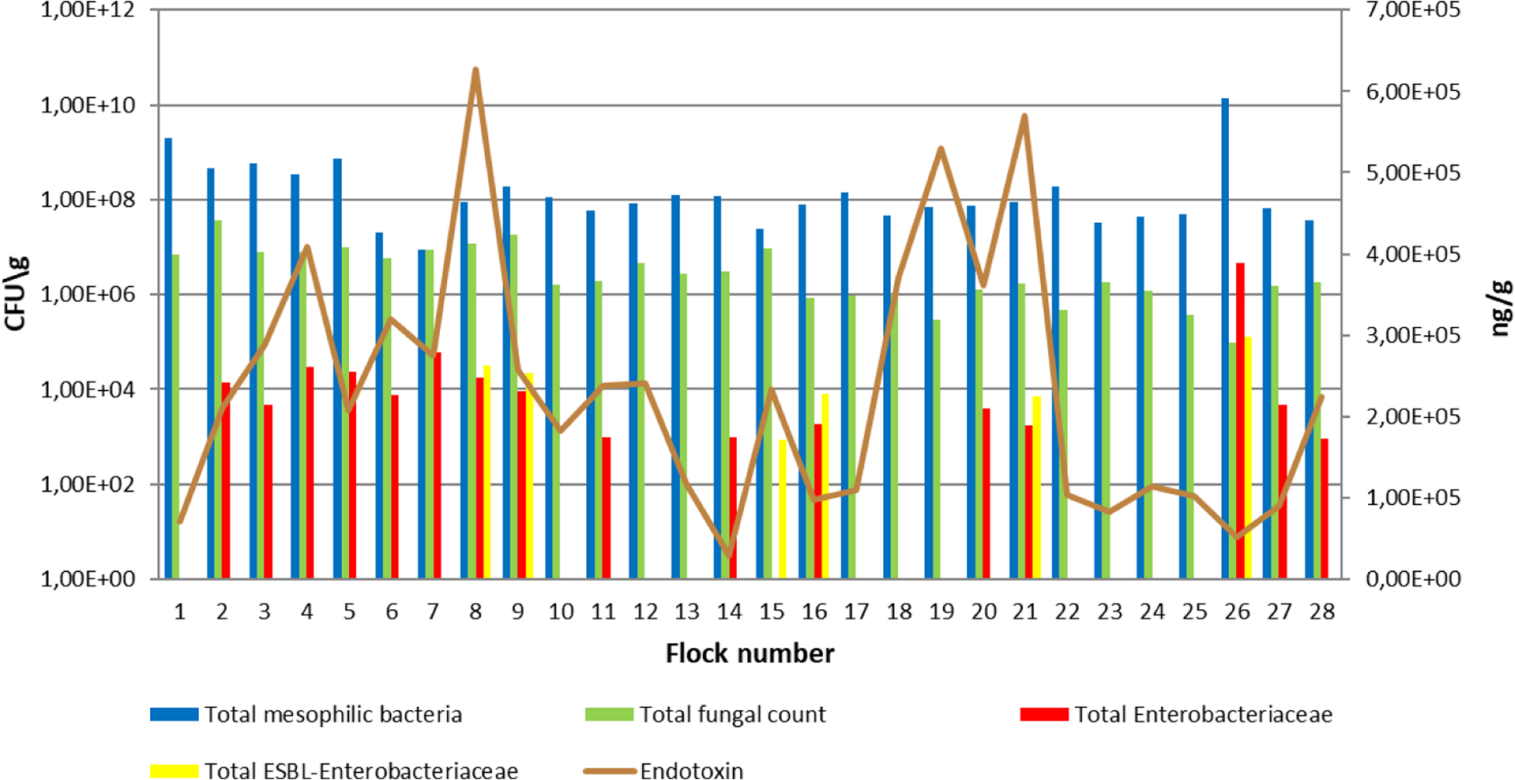
Total mesophilic bacteria, fungi, *Enterobacteriaceae*, ESBL-producing *Enterobacteriaceae*, and endotoxin concentrations in settled dust samples collected from laying hen farms (*n* = 28) after direct plating

All 28 pool samples showed the growth of total mesophilic bacteria and fungi (Fig. 1). Concerning the age of dust samples, no trends were observed, and no significant associations were found. The number of total mesophilic bacteria in settled dust samples varied from 9.07 × 10^6^ colony-forming units (CFU)/g in flock 7 (minimum) to 1.41 × 10^10^ CFU/g in flock 26 (maximum), with a mean ± standard deviation (SD) of 7.10 × 10^8^ ± 2.55 × 10^9^ CFU/g. Relative to the bacteria, the total fungal count was clearly lower (minimum of 9.84 × 10^4^ CFU/g in flock 26, maximum of 3.67 × 10^7^ CFU/g in flock 2, mean of 5.37 × 10^6^ ± 7.26 × 10^6^ CFU/g).

Neither *Salmonella* spp. nor MRSA were detected in dust samples (Table 1). The detection frequency of *Enterobacteriaceae* was 57% (16/28 samples) by direct plating. A particularly high number of these bacteria were detected in dust from flock 26 (4.65 × 10^6^ CFU/g). The lowest concentration (9.08 × 10^2^ CFU/g) was measured in flock 28 with a mean (1.72 × 10^5^ CFU/g) of more than three log steps below the number of the total bacteria count (Fig. 1). *Enterobacteriaceae* were detected more often by pre-enrichment (20/28 flocks) than by direct plating (Table 1). However, samples from three flocks [8, 9, 21] showed growth for *Enterobacteriaceae* with direct plating and no growth was observed after enrichment, resulting in a total of 23 positive samples that corresponded to a percentage of 82% positive flocks. In comparison to the *Enterobacteriaceae* results, ESBL-producing *Enterobacteriaceae* were less frequently detected by direct plating (21%, six positive samples). A high concentration of these bacteria was detected in dust from flock 26 (1.27 × 10^5^ CFU/g), while the lowest concentration (8.53 × 10^2^ CFU/g) was measured in the sample from flock 15 (Fig. 1). The mean of ESBL suspected isolates was 6.98 × 10^3^ CFU/g. Moreover, the detection level increased after enrichment to 16 positive samples (57%), including the positive samples from direct plating (Table 1). Additionally, ESBL-producing *Enterobacteriaceae* were detected in a further seven dust samples from *Enterobacteriaceae* isolates detected by direct plating on MacConkey agar (MacC) after spreading on Brilliance™ ESBL agar (Br-ESBL). Thus, in total, 23 dust samples (16 + 7) were primarily positive for ESBL-producing *Enterobacteriaceae.*

**Table 1:** Detection of *Enterobacteriaceae*^a^ and ESBL-producing *Enterobacteriaceae*^a^ and occurrence of MRSA and *Salmonella* spp. in settled dust from laying hen farms (*n* = 28)

	***Enterobacteriaceae*** recovered from MacC agar plate			ESBL ***Enterobacteriaceae*** recovered from Br-ESBL agar plate				MRSA on CHROM agar (PE)	***Salmonella*** spp. on MSRV Agar (PE)
	DP	PE	Total positive	DP	DP MacC	PE	Total positive		
**Positive samples**	16	20	23	6	7	16	23	0	0
**Percent (%)**	57	71	82	21	25	57	82	0	0

*MacC* MacConkey, *Br-ESBL* Brilliance Extended Spectrum Beta Lactamase, *MRSA* Methicillin-resistant *Staphylococcus aureus*, *MSRV* Modified Semi-solid Rappaport-Vassiliadis, *DP* Direct Plating, *PE* Pre-Enrichment

^a^After direct plating and/or enrichment in Luria-Bertani broth

The matrix-assisted laser desorption/ionization time of flight mass spectrometry (MALDI-TOF MS) results of collected isolates revealed that *E. coli* (*n* = 64) occurred in all the *Enterobacteriaceae* positive dust samples (23/23). *Enterobacter cloacae* (*E. cloacae*) (*n* = 20) was detected in almost half of the positive samples (12/23), while *Klebsiella pneumoniae* (*K. pneumonia*) (*n* = 16) was detected in lowest frequencies in the positive samples (6/23).

ESBL-producing *Enterobacteriaceae* isolates were confirmed by the broth microdilution test in 20 out of 28 dust samples (71%).

Endotoxins were detected in all samples. The concentrations varied from 2.9 × 10^4^ to 6.27 × 10^5^ ng/g, with an average of 2.23 × 10^5^. The detection of endotoxins was not related to the occurrence of cultivable *Enterobacteriaceae* (Fig. 1).

The detected values of temperature (°C) and relative humidity (%) are shown in Table 2. The relative humidity ranged between 50 and 71% without a clear trend. In contrast, the temperature was lowest in the winter period and highest in the summer period.

**Table 2:** Temperature (°C) and relative humidity (%) during the collection of settled dust in laying hen farms (*n* = 28) at different seasons

Farm	Season	Temperature (°C)	Relative humidity (%)
1	Summer	30.4	63.2
2	Summer	24.2	58.4
3	Summer	25.1	60.9
4	Summer	22.4	60.9
5	Summer	25.1	66.0
6	Fall	24.4	70.2
7	Fall	28.6	56.2
8	Fall	26.9	66.3
9	Fall	22.5	64.2
10	Fall	23.1	61.9
11	Fall	26.4	56.6
12	Winter	20.2	53.3
13	Winter	21.7	54.1
14	Winter	21.8	68.5
15	Winter	24.9	65.1
16	Spring	20.5	57.3
17	Spring	23.1	67.7
18	Spring	21.4	71.1
19	Spring	23.2	55.4
20	Spring	26.9	53.8
21	Spring	26.2	62.9
22	Spring	23.3	57.8
23	Spring	26.6	69.3
24	Summer	25.2	58.9
25	Summer	27.8	49.8
26	Summer	29.5	65.8
27	Summer	25.6	65.2
28	Summer	28.3	55.6

Correlation analysis revealed a low correlation between the total bacteria count and the age of stored samples (r_SP_ = 0.3959, *p* = 0.0370) (Table 3). The regression analysis showed that the total bacteria count was associated significantly with the age of stored samples only in the case of the concentrations of *Enterobacteriaceae* (*p* < 0.0001, F 41.25).

**Table 3:** Spearman’s rank correlation coefficient analysis between the total bacteria count and the measured variables^a^

	Fungi	***Enterobacteriaceae***	ESBL ***Enterobacteriaceae***	Endotoxins	Temperature (°C)	Humidity (%)	Age of dust sample
**r**_**SP**_	0.1472	0.3941	0.7143	−0.1888	−0.1388	0.0816	0.3959
***P*-Value**	0.4547	0.1309	0.1108	0.3359	0.4812	0.6799	0.0370

*r*_*SP*_ Spearman’s rank correlation coefficient

^a^fungal concentration, *Enterobacteriaceae* concentration, endotoxin concentration, temperature, humidity and age of the sample

## Discussion

Multiple studies from Europe have reported that sedimentation and airborne dust from broiler and turkey barns and layer hen houses can contain high microbial loads and endotoxin concentrations when compared to other farm animals, such as cattle or pigs [2, 23]. To the authors’ knowledge, there are few comparable studies for poultry farms in semi-arid regions. Therefore, the present study is the first to analyze microbial and endotoxin concentrations and the occurrence of resistant bacteria in dust from Egyptian laying hen houses. The only available data published in Egypt are about airborne culturable bacteria and fungi at one small-scale poultry farm [24]. This previous study found air contamination levels with 6.23 × 10^5^ CFU/m^3^ and 2.13 × 10^3^ CFU/m^3^ for bacteria and fungi, respectively [24]. Furthermore, another longitudinal study performed on three Egyptian broiler farms detected the fungal contamination level of settled dust with averages varying from 1.00 × 10^2^ CFU/g 1 day before introducing new chicks into the farm to 3.90 × 10^4^ CFU/g at the end of the fourth week of the production cycle [25]. The average total bacterial and fungal count recovered in the current study was 7.10 × 10^8^ CFU/g and 5.37 × 10^6^ CFU/g, respectively. Similar results were obtained from a Polish study in which the mean level of total bacteria in settled dust on three laying hen farms amounted to 9.60 × 10^8^ CFU/g and for the total fungal count to 2.08 × 10^6^ CFU/g [26]. Hartung and Saleh [2] counted 2.0 × 10^8^ to 1.7 × 10^9^ bacteria (CFU/g) and 4.0 × 10^5^ to 1.2 × 10^6^ fungi (CFU/g) in three different laying hen systems in Germany. The mean average temperatures during the daytime in laying hen houses in Northern Europe ranged between approximately 18 and 22 °C [27]. The mean temperature measured during the daytime in the presented study was 24.8 °C. The differences between the relative humidity measured in the study by Seedorf et al. [27] and that in our study were less clear. Concerning the temperature, there was no evidence that the number of cultivatable microorganisms in settled dust were mainly affected by climatic differences.

Multiple studies have shown that dust in laying hen houses can be a source of potential pathogens, zoonotic agents, resistant bacteria, and toxic compounds [1, 28]. *Salmonella* spp. (as important pathogens) and emerging resistant bacteria (such as MRSA and ESBL-producing bacteria) were selected as indicator organisms for potential hazards. *Salmonella* spp. can probably be spread by contaminated dust [29], and dust as an environmental sample is sufficient to detect *Salmonella* spp. in infected flocks [30]. Since no *Salmonella* spp. were detected in any of the 28 investigated flocks, the prevalence of *Salmonella* spp. on laying hen farms seems to be low in the Dakahlia province. However, the detection sensitivity on farms could be enhanced by using additional methods, including the investigation of eggs, feces or caeca of the birds [31]. Therefore, we could not state that laying hens in the regions are free from *Salmonella* spp.

Dust is also a sufficient source to detect MRSA in animal husbandries [32]. European studies have reported that 0.7 to 35% of poultry flocks, including laying hens, could be colonized with MRSA [33]. Different antibiotic managements in animal husbandries likely lead to lower MRSA prevalences in laying hens compared to broilers or turkeys [34]. Because of the frequent MRSA detection in diseased Egyptian broiler flocks [35, 36], this trend might be true for Egyptian poultry farms. However, no published data are available about the use of antibiotics in Egyptian poultry farms. Another fact is that the survival of LA-MRSA in the dust samples could have been influenced by the storage time. Although Schulz et al. [37] showed that LA-MRSA can stay viable at least for 17 months in poultry dust stored under comparable conditions (4 °C in the dark), the authors also showed that cultivability is lost during storage. Therefore, we cannot exclude that LA-MRSA occurred originally in the samples. More comprehensive and nationwide studies are necessary to describe the LA-MRSA prevalence in Egyptian poultry flocks.

There are few studies focusing on the level of *Enterobacteriaceae* in settled poultry dust. Two studies carried out in the Netherlands and Poland found that the mean of *E.coli* concentrations in settled dust from laying hen barns were 4.1 × 10^5^ and 1.6 × 10^5^ CFU/g, respectively [14, 26], which is comparable to the finding in this study (1.72 × 10^5^ CFU/g). Skora et al. [26] assumed an association between *E. coli* concentrations and viable bacterial counts. Our findings show a low correlation between total bacterial counts and *Enterobacteriaceae* concentrations (r_Sp_ = 0.3941, *p* = 0.1309), but a significant effect in the regression model. As most cultivable bacteria in dust are Gram-positive cocci [38, 39], this result could not have been expected since Gram-positive cocci are thought to be more resistant against environmental influences [40]. Furthermore, we found a correlation between the total bacteria counts and the age of the stored dust samples. These results are in accordance with previous investigations on the survival of *E. coli* in stored dust samples from broilers [20].

These results demonstrate a high occurrence of ESBL-producing *Enterobacteriaceae* (71%) in dust from Egyptian laying hen farms. It should be noted that clinical breakpoints were used for the classification of isolates; if epidemiological cut-off values were used, which are 1–2 dilution steps lower (e.g., according to EU Directive 2013/652/EU), the percentage would likely have been even higher. These are the first results on this topic from Egyptian laying hen holdings. Confirmed ESBL-producing *E. coli* were isolated from the dusts of 18 farms (64%). In a Dutch study, the authors found a total of 81 and 40% ESBL-producing *E.coli* positive dust samples collected from broilers and laying hens, respectively [14]. The use of antibiotics in laying hens in the present study was unknown. However, in Egypt, there is a lack of effective regulations regarding antibiotic use in animal husbandry for both the treatment and prevention of diseases, or, in many cases, as growth promoters, especially in the poultry sector [41]. This could explain the differences between the current study and the Dutch study, as the inappropriate use of antibiotics in both animals and humans is the main driver for the increase in multi-drug-resistant bacteria [42, 43]. Antibiotic-resistant bacteria in dust are most probably a result of fecal particles in the dust [20]. Their detection in dust means that the environment can be contaminated both by airborne emissions and by spreading manure.

The average detection level of ESBL-producing *Enterobacteriaceae* was 6.98 × 10^3^ CFU/g. This shows that ESBL-producing *Enterobacteriaceae* are only a small part of total culturable bacteria (7.10 × 10^8^ CFU/g), and concentrations can be below the detection limit of quantitative methods. It is known that the pre-enrichment of bacteria in environmental samples can lower the detection limit of ESBL-producing *Enterobacteriaceae* [44, 45]. In the present study, the pre-enrichment of bacteria in settled dust samples increased the detection rate of *Enterobacteriaceae* from 71 to 82% and for ESBL-producing *Enterobacteriaceae* from 21 to 57%. It seems that pre-enrichment is a useful method for more sensitive detection of resistant *Enterobacteriaceae* in dust from poultry houses. However, it should also be considered that AmpC-forming *E. coli* might not have been detected due to a pre-selection on Brilliance ESBL agar. However, AmpC-producing *E. coli* also show reduced susceptibility to cephalosporins, so that a higher proportion of isolates with resistance could still be present.

Many environmental investigations showed that poultry houses contain a higher amount of airborne microorganisms and endotoxin concentrations than other animal houses [23, 46]. The results of the presented study show that the settled dust in Egyptian laying hen houses was polluted with high levels of bacterial endotoxins. The median concentrations of endotoxin found in the samples of settled dust in the present study are similar to those reported in earlier studies [2]. Endotoxin concentrations did not correlate significantly with the total bacterial concentrations and *Enterobacteriaceae* concentrations. This can be explained by the fact that most bacteria in poultry house dust are Gram-positive and that endotoxins can be either from dead or viable but non-culturable bacteria or from Gram-negative bacteria of other families [47]. However, the concentration of airborne endotoxins was not investigated in the presented study. Nonetheless, high concentrations of airborne dust carrying endotoxins can be suspected in littered laying hen houses [48, 49]. Therefore, a considerable and potentially harmful exposure of farmers or other persons working routinely in laying hen houses can be expected [50]. To prevent negative health effects in humans by exposure to contaminated dust in laying hen farms, it is recommended to wear efficient dust masks while working in such farms. Negative effects on the health of laying hens by endotoxin exposure cannot be excluded [8], but conclusive studies on this have not yet been published.

## Conclusion

In conclusion, this study provides the first set of scientific data on ESBL-producing *Enterobacteriaceae*, as well as endotoxins and bacterial and fungal loads in settled dust from Egyptian laying hen houses. In the airborne state, dust might impair the health of birds and farmers. Furthermore, laying hens in Egypt seem to be a reservoir for ESBL-producing *Enterobacteriaceae*. Thus, farmers are at risk of exposure to ESBL-producing bacteria, and colonized hens might transmit these bacteria into the food chain. Prudent use of antibiotics and the implementation of good biosecurity practices in the primary sector are recommended to reduce the occurrence of ESBL-producing *Enterobacteriaceae* in Egyptian poultry houses. Studies about the use of antibiotics in poultry holdings would be helpful in analyzing associations between the occurrence of antibiotic-resistant bacteria and applied antibiotic agents.

## Methods

### Poultry farms

The studies were conducted at 28 laying hen farms in Dakahlia Province, Egypt, located in 31.0832° N, 31.4913° E latitude. The selection of farms was carried out randomly from different districts across Dakahlia province. The minimum distance between adjacent farms was 10 km. Farmers were contacted by phone maximum a week before sampling. None of the farmers cleaned the barn from dust during the laying period. One flock from each farm was sampled. If farms had more than one flock, the sampled flock was selected randomly. The age of hens during sampling ranged from 24 to 37 weeks. The settled dust samples were collected between the years 2016 and 2017. The hens in all barns were housed on a deep litter system, and the number of animals ranged from 3500 to 10,000 birds (Avian 48 or Hubbard Classic or Ross breed) per flock. Laying hen houses were either mechanically or tunnel ventilated. The feed and water were offered to the laying hens ad libitum.

### Dust collection

From each farm, one pooled sample of settled dust was collected from one flock. Settled dust was collected from ten different elevated surfaces inside the barn, including the drinking system line, feeding system line, and ventilation opening. Dust samples were collected in the morning between 09:00 and 10:00, by brushing settled dust from all sampling points into a sterile container with a sterilized brush. The samples were stored at 4 °C in the dark for 4 to 18 months in Mansoura University in Egypt before being analyzed retrospectively in a laboratory in Germany at the University of Veterinary Medicine Hannover, Foundation, within the framework of cooperation between Mansoura University and the University of Veterinary Medicine Hannover, Foundation. Dust samples were anonymized before sent to Germany.

### Microbiological analysis of dust

#### Enumeration of the total viable mesophilic bacteria and fungi

The total viable counts of aerobic mesophilic bacteria and fungi in dust samples were examined by plating dilution series from dust suspensions. Dust suspensions were prepared as described by Schulz et al. [20]. Briefly, 0.1 g dust was added to 10 mL phosphate buffer saline (PBS) with 0.01% TWEEN20 (v/v). Then, the suspension was shaken for 30 min in a water bath at 25 °C. Afterward, the suspension was vortexed for 4 min (Scientific Industries Inc., Bohemia, NY, USA). Aliquots (0.1 mL) of suspensions and serial dilutions from these suspensions were plated in triplicate on Tryptone Soya Agar plates (TSA, Oxoid Ltd., Basingstoke, UK) and on Dichloran-Glycerol (DG-18) agar supplemented with chloramphenicol (Oxoid Ltd) for counting colonies of mesophilic bacteria and fungi colonies, respectively. Negative controls were prepared by inoculating TSA and DG-18 media with 0.5 mL PBS. TSA plates were incubated for 48 h at 37 °C, while DG-18 agar plates were incubated at 25 °C for 5–7 d. After incubation, CFU was counted, and the results were expressed in CFU/g of dust.

#### Detection of *Salmonella* spp. and MRSA

For detecting *Salmonella* spp., an enrichment method was performed. Briefly, 1 mL dust suspension was added to 9 ml peptone water (PW, Oxoid Ltd) and incubated at 37 °C for 24 h. Thereafter, three aliquots of 100 μL each were added to the surface of Modified Semi-solid Rappaport-Vassiliadis (MSRV) agar (Oxoid Ltd) and incubated at 42 °C for 48 h. The growth of grey-white migrated cells with a turbid zone extending out from the inoculated drop was considered presumptively positive for *Salmonella* spp. Additionally, the detection of MRSA was also carried out by enrichment. One milliliter of dust suspension was added to 9 ml of Mueller-Hinton broth (MH, Oxoid Ltd) with 6.5% NaCl and incubated for 24 h at 37 °C. Following incubation, 1 ml of the MH suspension was added to 9 mL Tryptone Soy Broth (TSB, Oxoid Ltd) containing 75 mg/L aztreonam and 3.5 mg/L cefoxitin to grow MRSA aerobically at 37 °C for 17 h. Thereafter, 10 μL of TSB were streaked onto a selective agar CHROMagar MRSA (MAST Diagnostica GmbH, Reinfeld, Germany), and plates were incubated under aerobic conditions for 24 h at 37 °C. Suspected colonies with pink or mauve coloration were considered to be MRSA. MRSA strain DSM 1182 (Leibniz-Institute DSMZ German Collection of Microorganisms and Cell Culture, Brunswick, Germany) and *Salmonella enterica subsp. enterica* serovar Livingstone isolates from the bacteria collection of the Institute for Animal Hygiene, Animal Welfare and Farm Animal Behaviour, University of Veterinary Medicine Hannover, Germany were used as growing controls.

#### Isolation and enumeration of *Enterobacteriaceae* and ESBL-producing *Enterobacteriaceae*

Aliquots (0.1 mL) from the original dust suspension and from a 10-fold and 100-fold dilution were plated in triplicate on Br-ESBL agar (Oxoid Ltd) and on MacC agar (Oxoid Ltd) to determine the count of ESBL-producing *Enterobacteriaceae* and also non-resistant *Enterobacteriaceae*, respectively. Plates were incubated at 37 °C for 48 h. Suspected ESBL-producing *Enterobacteriaceae* and *Enterobacteriaceae* colonies were counted, and the CFU per gram of dust was calculated. For recovering environmentally stressed ESBL-producing *Enterobacteriaceae* and *Enterobacteriaceae*, pre-enrichment of 1 mL dust suspension was added to 9 ml Luria-Bertani broth (LB, Oxoid Ltd) and incubated at 37 °C for 24 h [44]. After incubation, 10 μL of the LB broth were streaked onto Br-ESBL and MacC agar in duplicate. Plates were incubated for 24 h at 37 °C and different *Enterobacteriaceae* suspected colonies (two to six different colonies, depending on the different appearance on the media) from each media were purified on Columbia blood agar (Oxoid Ltd) and stored in cryovials (CRYOBANK™, MAST Diagnostica GmbH, Germany) at − 80 °C for further identification. Negative controls were prepared by inoculating MacC and Br-ESBL media with 0.5 mL PBS. As growing controls, ESBL-*E. coli* strain DSM 22664, ESBL-*K. pneumoniae* strain DSM (26371), and *E. coli* DSM 1103 (Leibniz-Institute DSMZ German Collection of Microorganisms and Cell Culture, Brunswick, Germany) were streaked onto Br-ESBL agar. Controls were incubated simultaneously with the samples.

When the dust sample showed *Enterobacteriaceae* colonies only on MacC agar, either directly or after enrichment but not on Br-ESBL media, the recovered *Enterobacteriaceae* colonies from MacC agar were further streaked onto Br-ESBL media and incubated as described above. Recovered *Enterobacteriaceae* colonies were also purified and stored for further analysis.

#### Identification of *Enterobacteriaceae*

Different *Enterobacteriaceae* isolates (three to five colonies from each sample), depending on growth characteristics, obtained after direct plating and/or after pre-enrichment from MacC and Br-ESBL media, were purified and further identified by MALDI-TOF MS (Bruker Daltonik GmbH, Bremen, Germany). Sample preparation was carried out in accordance with the protocols provided by the manufacturer using the direct transfer method.

#### ESBL-producing *Enterobacteriaceae* determination

*Enterobacteriaceae* isolated from dust samples were assigned to ESBL phenotypes by the broth microdilution method following the Clinical and Laboratory Standards Institute (CLSI) document VET01S (CLSI, 2015). Screening of isolates was performed with ceftazidime and cefotaxime at concentrations ranging from 1 to 128 μg/mL. Growth at 1 μg/mL or above this antibiotic concentration is suspicious for ESBL production and precondition for a phenotypic confirmatory test. For these phenotypic tests, commercially available microtiter plates (MERLIN Gesellschaft für Mikrobiologische Diagnostika GmbH, Bornheim-Hersel, Germany) were used. The plates contained the following antibiotics and concentrations in two-fold dilution steps: cefepime (CEP) (1–128 μg/mL), cefepime plus clavulanic acid (CMC) (0.25/4–32/4 μg/mL), ceftazidime (CAZ) (1–128 μg/mL), ceftazidime plus clavulanic acid (CZ (0.25/4–32/4 μg/mL), cefotaxime (CTX) (1–128 μg/mL), and cefotaxime plus clavulanic acid (C/C) (0.25/4–32/4 μg/mL). Isolates were considered ESBL-producing positive when the differences in the minimal inhibitory concentration values (MIC values) of cefepime, ceftazidime, and cefotaxime tested individually and in combination with clavulanic acid were greater than or equal to three dilution steps. Quality controls were performed by testing a non-ESBL-producing *E.coli* (DSM 22664) and an ESBL-producing *K. pneumonia* (DSM 26371) quality control strain.

### Endotoxin levels in dust

One milliliter of a 10-fold diluted dust suspension was stored in a tube at − 80 °C until analysis for determining the endotoxin concentrations. The endotoxin concentrations were measured with the Limulus Amebocyte Lysate test (LAL-test, Biowhittaker Inc., Walkersville, MD, USA) in accordance with a standardized chromogen-kinetic procedure (Kinetic-QCL, Lonza Group AG, Basel, Switzerland). Only sterile and pyrogen-free glassware and microplates (Greiner Bio-One International GmbH, Frickenhausen, Germany) were used. The control endotoxin standard (*E. coli* O55:B5) was diluted to 50, 5, 0.5, 0.05, and 0.005 EU/mL to create a calibration curve. The endotoxin activity was indicated in endotoxin units (EU) and calculated to ng/g in accordance with the manufactures instructions.

### Temperature and humidity

Air temperature (°C) and relative humidity (RH%) were measured by a Digital thermo-hygrometer (Xuzhou Sanhe Automatic Control Equipment Co., Ltd., Xuzhou, China). Values were recorded during the dust collection.

### Statistical analyses

Statistical analyses were performed using the SAS 9.4 software (SAS Institute Inc., Cary, NC, USA). Correlations between the total bacteria count and measured variables (fungal concentration, *Enterobacteriaceae* concentration, endotoxin concentration, temperature, humidity, and age of sample) were tested using the Spearman’s rank correlation coefficient. The chosen variables might directly affect the survival of total bacteria in dust or are potentially closely associated with their count. A linear regression was performed to investigate significant associations of the variables with the total bacteria count. The level of significance was set at *p* = 0.05.

## Data Availability

The datasets used and analyzed during the current study are available from the corresponding author on reasonable request.

## Abbreviations

CFU: Colony-forming units
spp.: Species
ESBL: Extended-spectrum beta-lactamase
LA-MRSA: Livestock-associated methicillin-resistant *Staphylococcus aureus*
*E. coli*: *Escherichia coli*
*E. cloacae*: *Enterobacter cloacae*
*K. pneumoniae*: *Klebsiella pneumoniae*
MALDI-TOF MS: matrix-assisted laser desorption/ionization time of flight mass spectrometry
SD: Standard deviation
MacC: MacConkey agar
Br-ESBL: BrillianceTM ESBL agar
PBS: Phosphate buffer saline
TSA: Tryptone Soya agar
DG-18: Dichloran-Glycerol agar
PW: Peptone water
MSRV: Modified Semi-solid Rappaport-Vassiliadis
MH: Mueller-Hinton
TSB: Tryptone Soy Broth
DSM: German Collection of Microorganisms
LB: Luria-Bertani
CLSI: Clinical and Laboratory Standards Institute
CEP: Cefepime
CMC: Cefepime plus clavulanic acid
CAZ: Ceftazidime
CZ: Ceftazidime plus clavulanic acid
CTX: Cefotaxime
C/C: Cefotaxime plus clavulanic acid
MIC: Minimal inhibitory concentration
LAL: Limulus Amebocyte Lysate test
EU: Endotoxin units
RH: Relative humidity
